# Functional Activation during the Rapid Visual Information Processing Task in a Middle Aged Cohort: An fMRI Study

**DOI:** 10.1371/journal.pone.0138994

**Published:** 2015-10-21

**Authors:** Chris Neale, Patrick Johnston, Matthew Hughes, Andrew Scholey

**Affiliations:** 1 Centre for Human Psychopharmacology, Swinburne University, Melbourne, VIC, 3122, Australia; 2 Stockholm Environment Institute, University of York, York, YO10 5DD, England; 3 School of Psychology, University of York, York, YO10 5DD, England; 4 School of Psychology & Counselling, Queensland University of Technology, Brisbane, Queensland, Australia; University Medical Center Goettingen, GERMANY

## Abstract

The Rapid Visual Information Processing (RVIP) task, a serial discrimination task where task performance believed to reflect sustained attention capabilities, is widely used in behavioural research and increasingly in neuroimaging studies. To date, functional neuroimaging research into the RVIP has been undertaken using block analyses, reflecting the sustained processing involved in the task, but not necessarily the transient processes associated with individual trial performance. Furthermore, this research has been limited to young cohorts. This study assessed the behavioural and functional magnetic resonance imaging (fMRI) outcomes of the RVIP task using both block and event-related analyses in a healthy middle aged cohort (mean age = 53.56 years, n = 16). The results show that the version of the RVIP used here is sensitive to changes in attentional demand processes with participants achieving a 43% accuracy hit rate in the experimental task compared with 96% accuracy in the control task. As shown by previous research, the block analysis revealed an increase in activation in a network of frontal, parietal, occipital and cerebellar regions. The event related analysis showed a similar network of activation, seemingly omitting regions involved in the processing of the task (as shown in the block analysis), such as occipital areas and the thalamus, providing an indication of a network of regions involved in correct trial performance. Frontal (superior and inferior frontal gryi), parietal (precuenus, inferior parietal lobe) and cerebellar regions were shown to be active in both the block and event-related analyses, suggesting their importance in sustained attention/vigilance. These networks and the differences between them are discussed in detail, as well as implications for future research in middle aged cohorts.

## Objectives

The Rapid Visual Information Processing task (RVIP) is a serial detection task devised by Bakan [1] that is used to probe visual sustained attention and working memory processes. Sustained attention, defined also as vigilance [2], is generally associated with ascending cholinergic systems originating in basal forebrain and extending to the cortex [3–5]. As such the RVIP has been used extensively in psychopharmacological studies of the cholinergic system [6–9]. Further, an ascending noradrenergic pathway originating in the locus coeruleus is believed to mediate the effects of arousal on sustained attention [2, 10].

The design of the task comprises of a stream of single digits (Arabic numerals 1–9) presented at a rate of 100–110 per minute. Participants are required to detect targets comprising a sequence of three consecutive odd or three consecutive even numbers [7, 11]. In a typical paradigm, targets are presented at a rate of four per 30 seconds [12–14]. The critical performance variables include the number of correct responses (‘hits’), the reaction time for hits, and the number of errors of commission (response in absence of a target). In neuroimaging paradigms, activation to the RVIP task is contrasted with that to a control task where participants see a stream of digits presented at the same rate, but are required to respond to a ‘0’ instead of the three consecutive odd or even numbers [12, 13]. Therefore, both the RVIP task and control task require sustained attention, stimulus discrimination and target responding for successful completion. However, in the RVIP task, participants must further recruit working memory processes to ensure that the correct target (the third consecutive odd or even number) is identified. The increased used of working memory, or attention [15–17], is what discriminates these two tasks.

The first neuroimaging study looking at RVIP [12] used positron emission tomography (PET) and showed, when compared with rest, task-related bilateral increases in regional cerebral blood flow (rCBF) in the inferior frontal gyrus, parietal cortex, and supplementary motor area (SMA) and in the right rostral superior frontal gyrus. These increases in activation in the right inferior and superior frontal gyri were not apparent when comparing the experimental task with a control condition. Further research has assessed the neural mechanisms of the RVIP task using functional magnetic resonance imaging (fMRI) methods. The task-related blood oxygen level dependent (BOLD) signal increases in right frontal, including the dorsolateral prefrontal cortex, and bilateral parietal regions are shown across studies [13, 14, 18, 19] as well as bilaterally in the thalamus [13, 14], right fusiform gyrus [13] and bilaterally in the cerebellum [13, 14, 18, 19]. BOLD deactivations have been shown in the angular gyrus, cuneus, precuneus, posterior cingulate and ventromedial prefrontal cortex during the experimental task compared with the control task [18]. This suggests that these regions are important in the processing of task relevant information and the execution of the RVIP experimental task. Three placebo-controlled fMRI studies also confirm that the RVIP task in its modified design, (i.e. including a control task), is sensitive to the effects of different doses and administration routes of nicotine in healthy participants [13, 18] and in schizophrenic individuals [19].

These neuroimaging studies have examined functional data only in the context of blocked fMRI designs. These designs are most sensitive to sustained brain processes that are active over the duration of task performance (i.e. performance of a block of trials), but are less sensitive to phasic brain processes that are engaged only during task trial performance. Additionally, since they have all used young cohorts, it is not clear, given the changes to brain with normal ageing, that these activation patterns also generalise to older participants, who might employ different strategies or engage a wider or more restricted brain network in performing the same task. Previous research has suggested that there are potential age-related changes in haemodynamic function and brain morphology [20, 21] as well as declines in attentional performance [22].

The aim of the current study was to examine both sustained and phasic brain processes that are engaged for RVIP task performance in middle-aged participants by utilising both blocked and event-related analyses. To assess the effects of sustained attention and possible fatigue effects, we also examine the activation between individual experimental and between individual control blocks in each scan session. We hypothesise that the neuroimaging data presented in the block analysis will reflect the distinct differences in cognitive processing required to complete the experimental task. Given the reliance of successful task completion on attention and working memory faculties, we expect that there will be increases in activation in the frontal (dorsolateral prefrontal cortex) and parietal lobes underpinning these processes. These regions of activity have been shown in previous RVIP imaging research[13, 14, 18, 19].

In terms of the behavioural results, we hypothesised that participants would perform at a qualitatively lower level on the task than previous research using young participants based on expected declines in attentional performance in an older cohort. Participants will show higher accuracy and faster reaction times in the control task and make less errors of commission when compared with the experimental task. This will be due to the relative ease of the control task when compared with the increased difficulty of completing the experimental task.

Given this study is the first in RVIP research, to the authors’ knowledge, to utilise an event related analysis, there is no precedent for expected outcomes. However, we anticipated that a similar network of activation would be present in the event-related analysis; in particular, recruitment of frontal cortex given its role in working memory. The event-related analysis assesses the processes concerned with response to the task, so may omit regions associated with general task processing when compared with the block analysis images.

## Methods

### Participants

Participants were right handed, healthy adults aged 41–64 years (N = 16, 6 male, 10 female, mean age = 53.56 years, standard deviation = 7.13) with normal or corrected-to-normal vision. Exclusion criteria for study participation included a history of anxiety, depressive, epileptic or psychiatric disorders, in addition to normal MRI contraindications that were screened for by the experimenters and radiography staff at the Brain Research Institute (Austin Hospital, Heidelberg, VIC, Australia). The project was approved by the Swinburne University Human Research Ethics Committee and conducted in concordance with the declaration of Helsinki. Written informed consent was obtained from all participants.

### Experimental Design and Procedures

Participants attended two sessions that included an initial practice session conducted at the Centre for Human Psychopharmacology (Hawthorn, VIC, Australia) and a subsequent experimental fMRI scanning session conducted at the Brain Research Institute (Austin Hospital, Heidelberg, VIC, Australia). During the practice session participants were briefed and trained on the task used during the experimental session (see below). Prior to the experimental session participants were given further practice on each task, then performed the tasks while undergoing fMRI scanning immediately afterwards.

All aspects of the task were controlled by Presentation^®^ software (Version 14.5, www.neuro-bs.com). All stimuli was presented visually on a computer monitor and viewed via a mirror mounted on the head coil.

The RVIP task was operationalised in a blocked fMRI design that included two separate scanning runs. Each scanning run contained six activation blocks each lasting sixty seconds in addition to seven rest periods each lasting twelve seconds that preceded and followed activation blocks. Each scanning run lasted 7 minutes and 24 seconds, therefore total task scanning time was 14 minutes and 48 seconds.

**Fig 1:**
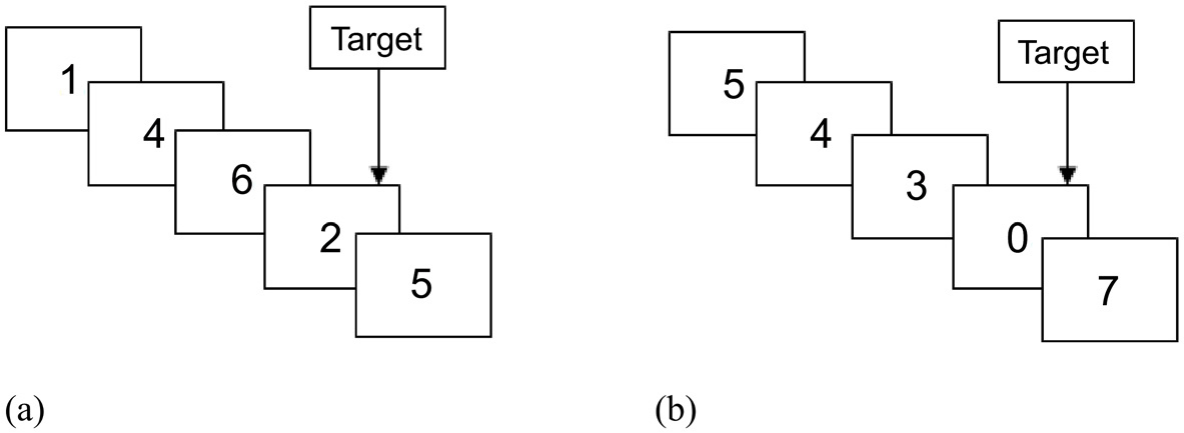
(a) Example of target response in experimental task block (b) Example of target response in control block.

Activation blocks were sub-divided into ‘Experimental task’ blocks and ‘Control task’ blocks (three blocks of each task per scanning run). Experimental task stimuli were the numerals 1–9 while Control task stimuli were the numerals 0–9, all presented in white font on a black background. All stimuli were presented pseudo-randomly and with equal-probability, and with an onset-to-onset interval and duration of 600 ms with no inter-stimulus interval. For the Experimental task, participants were asked to respond by right hand, index finger button-press when they saw three consecutive odd numbers or three consecutive even digits (‘triplets’) appear in any sequence (e.g. 3-5-7, 8-2-4; see Fig 1). Control task blocks did not contain triplets; participants were asked to respond whenever they saw a ‘0’. To ensure that Control task and Experimental task blocks were matched for motor activation, both block types exhibited eight targets (‘0’ or odd/even triplets) appearing at a rate of 4 per 30 seconds. In the experimental task, ‘Target Correct’ refers to the number of odd or even triplets the participant successfully identified, whilst in the control task, it refers to the number of zeroes correctly identified. Errors of commission refer to trials where participants have responded to seeing a target in the absence of a target.

Between each block was a twelve second rest period which also contained prompts that informed participants what type of activation block followed (Experimental/Control). The prompts read either ‘zeroes’ to indicate the Control task block (C), or ‘odds/evens’ to indicate an Experimental task block (E).

The order of block presentation was always C-E-C-E-C-E. Four different versions of the task were created for randomisation with one version being repeated twice during the same scan session for each participant. A Latin square was used to assign a task version to the participant’s visit, ensuring that task version was balanced across participants.

### fMRI Acquisition

FMRI scanning was performed using a Siemens Tim Trio 3T MRI scanner with a 12-channel head coil at the Brain Research Institute, Heidelberg, Australia. Initially, a high-resolution structural image was acquired using a T1 MPRAGE sequence with 192 sagittal slices and 0.9mm isometric voxels (TR = 1900ms, TE = 2.6ms, flip angle = 9 degrees), for later registration of functional images into standard stereotactic (MNI) space. During the functional scanning runs 145 images were acquired using a gradient-echo, echo-planar T2*-weighted sequence (TR = 3000 ms; TE = 30 ms; flip angle = 85 degrees; field of view = 216 x 216mm; 72 x 72 imaging matrix). The commencement of the functional paradigm was triggered by the onset of the 4^th^ volume and volumes 1 through 3 were discarded to negate T1 saturation effects in EPI images. Each image comprised of 44 3-millimetre slices acquired approximately axially in an interleaved order, with an in-plane resolution of 3 x 3 mm and no slice gap.

### fMRI Analysis

#### Image Pre-processing

Preprocessing and statistical analyses were performed using SPM8 [23, 24] and associated toolboxes. For the block analysis, the ArtRepair routines [25] were used to denoise voxels and then repair highly variant slices. The corrected images were then realigned to the first volume, and a mean image was created. The T1 image was co-registered to the mean EPI, then normalised to the T1 template supplied with SPM8. The parameters of this T1 transformation were subsequently applied to the realigned functional images. The normalised functional images were then smoothed with an 8mm FWHM Gaussian filter. The final voxel size was 2 x 2 x 2 mm. Finally, ArtRepair was used to repair any volume outliers in individual data sets.

Preprocessing for the event related analysis followed the same steps as above, but prior to image realignment, correction for the acquisition delay between slices (slice time correction) was applied using the first slice as the reference.

### Statistical Analysis

#### Participant Level Modelling

Image time-series data were modelled as both a block-design and an event-related design. In the block design model, experimental and control blocks were modelled using a separate boxcar function defined by the onset and duration of each block convolved with the SPM8 canonical haemodynamic response function. Image realignment parameters were added to the model as regressors of no interest (6 regressors; 3 rotational parameters and 3 translational parameters) to account for movement correlated BOLD signal variance as well as using a 217 second high-pass filter. After model estimation, the contrasts of Experimental > Control and Control > Experimental were computed and entered into group level models.

To assess the temporal activation differences within each condition, we compared the activation between individual blocks of the same type (e.g. experimental block one vs. experimental block two vs. experimental block three). Individual blocks were modelled using the same general linear model as above using different contrasts. Both individual experimental blocks and control blocks were combined from each of the two scanning runs within the scanning session (i.e. experimental block 1 is equal to the mean of experimental block 1 over the two scanning runs). The steps from the initial block design analysis were then undertaken comparing between each block within a particular block type.

In the event-related model, separate regressors defining ‘hits’ during each of the experimental task and control task blocks as well as regressors for errors of commission were modelled as events (duration 0ms). As with the block-design analysis, the realignment parameters were added to model head movement. The contrast of Experimental Hits > Control Hits and Control Hits > Experimental Hits as well as Experimental Hits > Errors of Commission and Errors of Commission > Experimental Hits were computed and subsequently used in group level analysis.

#### Group Level Modelling

Group effects for both the block and event-related analyses were assessed by entering contrasts of interest into random effects one-sample t-tests. Further analysis on the event-related contrasts also included a record of overall task performance as a covariate in order to establish if differences in activation between the activation in the control and experimental tasks was merely due to differences in task difficulty, as opposed to the processes underpinning the task. Overall task performance was defined as the mean hits during the experimental and control tasks and this data was inputted into a 2^nd^ Level SPM results script. These activation maps at voxel level were thresholded at p < .001 (uncorrected) then corrected for multiple comparisons at cluster level using an extent threshold that excluded non-significant clusters (p < .05 Family-Wise Error (FWE) correct).

The data for the contrasts described in this manuscript as well as the raw data can be found stored in the Harvard Dataverse. The link to this is http://dx.doi.org/10.7910/DVN/12BLHZ.

## Results

### Behavioural Results

Participants performed near ceiling level on the control task but were less accurate in identifying targets in the experimental task as shown in Table 1. Reaction times were slower in the experimental task than in the control task. In both measures of accuracy and reaction time, the experimental task showed more variability than the control task. There were false positives seen in the experimental task, but not in the control task.

**Table 1:** Behavioural results for the individual components of the RVIP task (experimental and control task). Mean scores with standard deviations in parentheses.

	Target Correct (/48)	Target Reaction Time (ms)	False Positives
Control task	44.19 (2.97)	485.77 (60.04)	0 (0)
Experimental task	20.63 (8.89)	553.34 (73.82)	4.94 (4.86)

Paired t-tests between experimental and control measures showed a significant difference across all three measures; target detection, t (15) = 10.10, p < .001, reaction time, t (15) = 3.83, p < .001 and false positives, t (15) = 4.06, p < .001.

To more accurately assess sustained attention effects, the behavioural data were assessed on a block-by-block basis, hence factoring time into the performance measures (see Table 2).

**Table 2:** Behavioural results for the individual components of the RVIP task (experimental and control task) divided by individual blocks. Mean scores shown with standard deviations in parentheses.

Block	Target Correct (/16)	Target Reaction Time (ms)	False Positives
C1	14.69 (1.14)	473.66 (57.34)	0 (0)
C2	14.06 (1.91)	488.62 (63.07)	0 (0)
C3	15.44 (0.89)	494.24 (65.04)	0 (0)
E1	6.44 (3.39)	547.50 (145.11)	1.81 (2.07)
E2	7.31 (3.83)	513.38 (159.83)	1.56 (2.25)
E3	6.88 (2.83)	492.11 (117.98)	1.38 (1.09)

C = Control Block,

E = Experimental Block.

The mean results indicate there was little variation in the number of targets correctly identified between blocks in both the control task and experimental task conditions. Notably however, reaction times appear to increase as a function of time on the control task, whereas reaction times appear to decrease as a function of time in the experimental task. However, these are qualitative observations and were not statistically significant. No false positives were recorded in the control task and little change is apparent for the number of false positives incurred in the experimental task. These means were further investigated using multiple repeated measures ANOVAs on ‘Target Correct’, ‘Reaction Time’ and ‘False Positive’ variables. There were no statistical differences between any of these measures across time.

### Neuroimaging Data

#### Block Analysis

**Fig 2:**
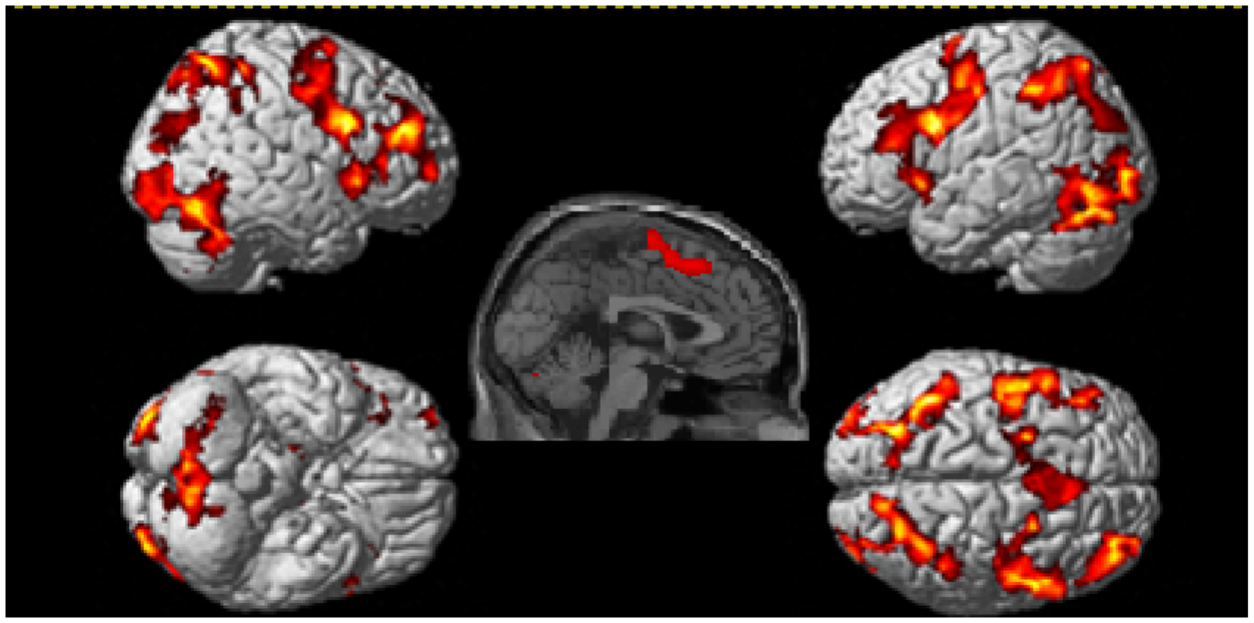
Brain regions active during the experimental task > control task contrast. Clusters were considered significant at a voxel height of p < .001 and cluster corrected at p < .05 (FWE corrected).

**Fig 3:**
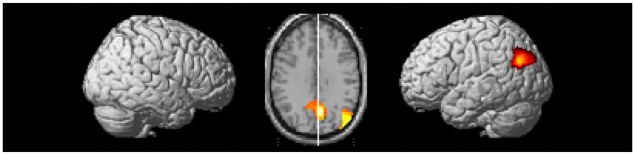
Brain regions active during the control task > experimental task contrast. Clusters were considered significant at a voxel height of p < .001 and cluster corrected at p < .05 (FWE corrected).

Whole brain analyses revealed six clusters of brain activation that differed significantly between the experimental and control conditions, four in the experimental > control contrast (see Table 3 and Fig 2) and two in the control > experimental contrast (see Table 4 and Fig 3). In experimental > control, BOLD increases were observed bilaterally in frontal, occipital, parietal areas and cerebellar cortical areas. All cluster regions in the following tables refer to the area of peak activation. The control > experimental contrast revealed increased activation in the temporal, inferior parietal and occipital lobes in the left hemisphere, with the peak activation shown in the temporal lobe as shown in the render and sagittal slice in Fig 2.

**Table 3:** Clusters showing differences in activation between the RVIP experimental task > control task (p = .001).

*Brain Region*	*Laterality*	*Cluster Size*	*Cluster Sig*.	*Peak t-score*	*MNI co-ords*
Middle Frontal Gyrus	L	6541	< .001	16.38	-44 2 38
Middle Frontal Gyrus	R	5407	< .001	10.87	48 6 36
Superior Parietal Lobule	R	13375	< .001	9.44	32–62 44
*Sub Region*: *Inferior Parietal Lobule*				9.01	40–54 50
Lentiform Nucleus	R	414	.034	5.35	24–14 2
*Sub Region*: *Thalamus*				4.48	20–8 16

MNI = Montreal Neurological Institute,

B = Bilateral,

L = Left Hemisphere,

R = Right Hemisphere,

Cluster Sig. = Cluster significance.

**Table 4:** Clusters showing differences in activation between the RVIP control task > experimental task (p = .001).

*Brain Region*	*Laterality*	*Cluster Size*	*Cluster Sig*.	*Peak t-score*	*MNI co-ords*
Super Occipital Gyrus	L	721	.004	5.19	-42–84 36
*Sub Region*: *Angular Gyrus*				5.16	-48–78 38
Cuneus	B	774	.003	4.85	-8–64 30
*Sub Region*: *Cingulate*				3.55	6–54 28

MNI = Montreal Neurological Institute,

B = Bilateral,

L = Left Hemisphere,

R = Right Hemisphere,

Cluster Sig. = Cluster significance.

T-contrasts were carried out between the control blocks and the experimental task blocks (i.e. control block one > control block two, experimental task block one > experimental task block three) with twelve contrasts used overall. There were no statistically significant clusters between any of the contrasts.

**Fig 4:**
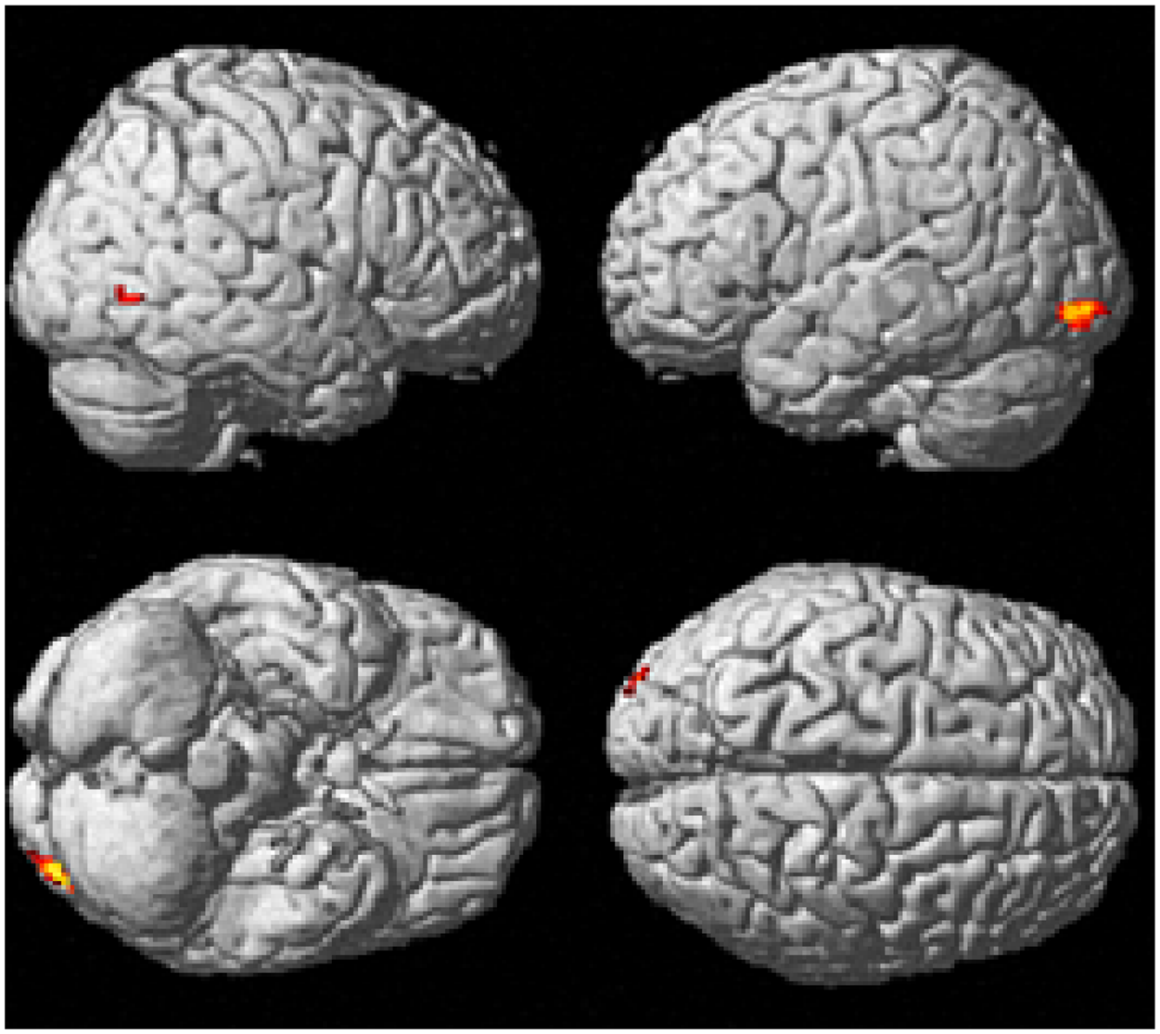
Brain regions active during the control task > baseline contrast. Clusters were considered significant at a voxel height of p < .001 and cluster corrected at p < .05 (FWE corrected).

Whole brain analyses reveal two clusters of brain activation that differed significantly between the control task and baseline (control task > baseline contrast). These clusters were defined as the left inferior occipital gyrus and the right inferior temporal gyrus. These regions can be seen in Table 5 and Fig 4.

**Table 5:** Clusters showing differences in activation between the RVIP control task > baseline (p = .001).

*Brain Region*	*Laterality*	*Cluster Size*	*Cluster Sig*.	*Peak t-score*	*MNI co-ords*
Inferior Occipital Gyrus	L	92	< .001	10.97	-34–90–6
Inferior Temporal Gyrus	R	22	< .001	7.68	44–66–4

MNI = Montreal Neurological Institute,

B = Bilateral,

L = Left Hemisphere,

R = Right Hemisphere,

Cluster Sig. = Cluster significance.

**Fig 5:**
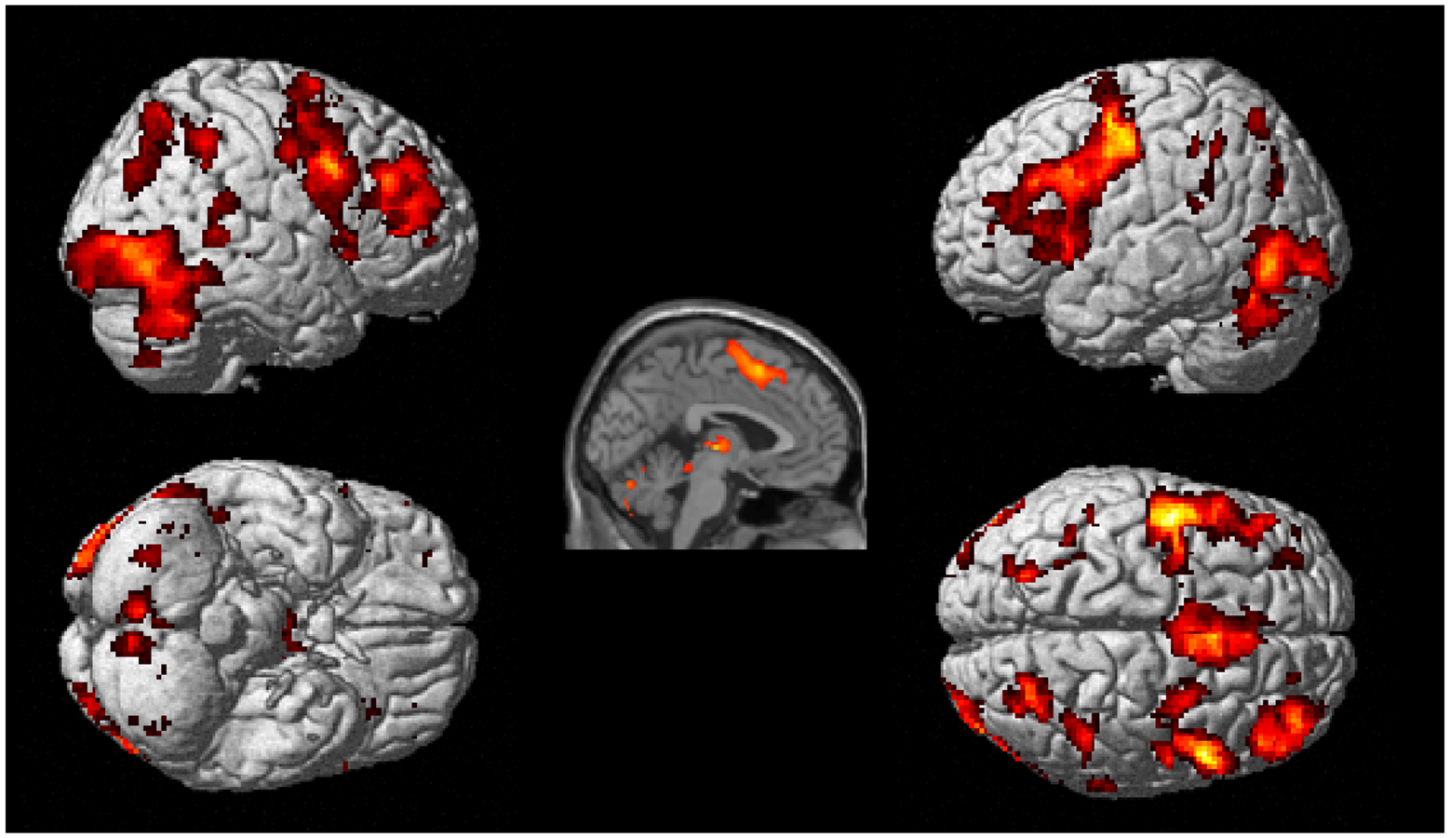
Brain regions active during the experimental task > baseline contrast. Clusters were considered significant at a voxel height of p < .001 and cluster corrected at p < .05 (FWE corrected).

Whole brain analyses revealed four clusters of brain activation that differed significantly between the experimental task and baseline (experimental task > baseline contrast). These clusters of activation were shown including occipital, cerebellar, parietal, and frontal regions. These regions can be seen in Table 6 and Fig 5.

**Table 6:** Clusters showing differences in activation between the RVIP experimental task > baseline (p = .001).

*Brain Region*	*Laterality*	*Cluster Size*	*Cluster Sig*.	*Peak t-score*	*MNI co-ords*
Precentral Gyrus	B	16380	< .001	14.82	-52–4 54
*Sub region*: *Cerebellum*				11.85	26–62–30
Precuneus	R	1123	< .001	12.24	30–72 32
*Sub Region*: *Inferior Parietal Lobe*				7.70	44–44 42
Cerebellum	L	1997	< .001	10.83	-30–64–22
Precuneus	L	551	< .001	9.50	-24–68 50

MNI = Montreal Neurological Institute,

B = Bilateral,

L = Left Hemisphere,

R = Right Hemisphere,

Cluster Sig. = Cluster significance.

**Fig 6:**
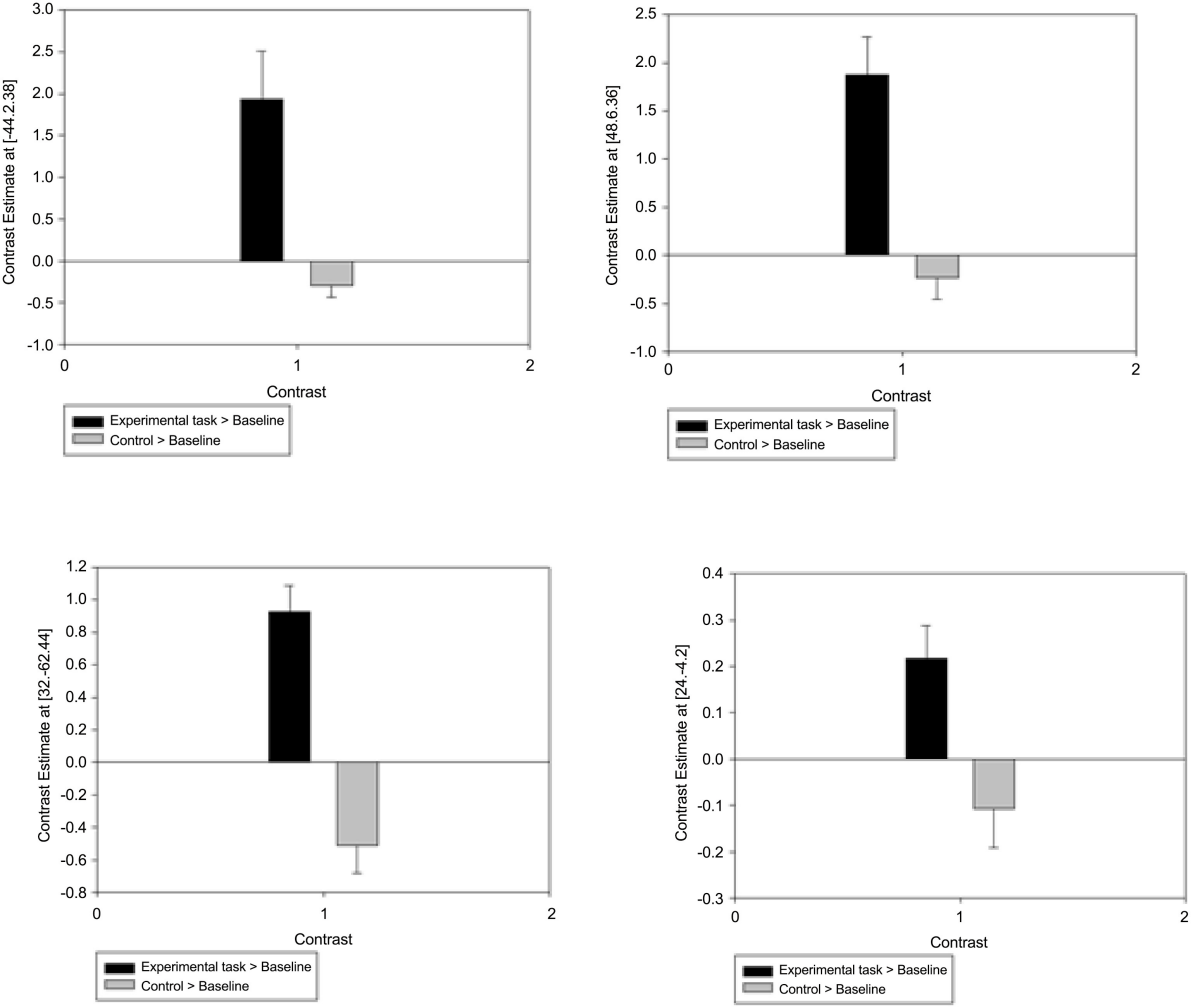
Contrast estimates (and standard errors) for experimental task and control task > baseline activation within the four significant clusters in the experimental task > control task contrast.

To contextualise the experimental and control task activation in comparison with baseline, Fig 6 shows the contrast estimates for both the experimental task > baseline and control task > baseline contrasts within the four significant clusters shown in the experimental task > control contrast (precentral gyrus, cerebellum and the right and left precuenus).

#### Event-related Analysis

**Fig 7:**
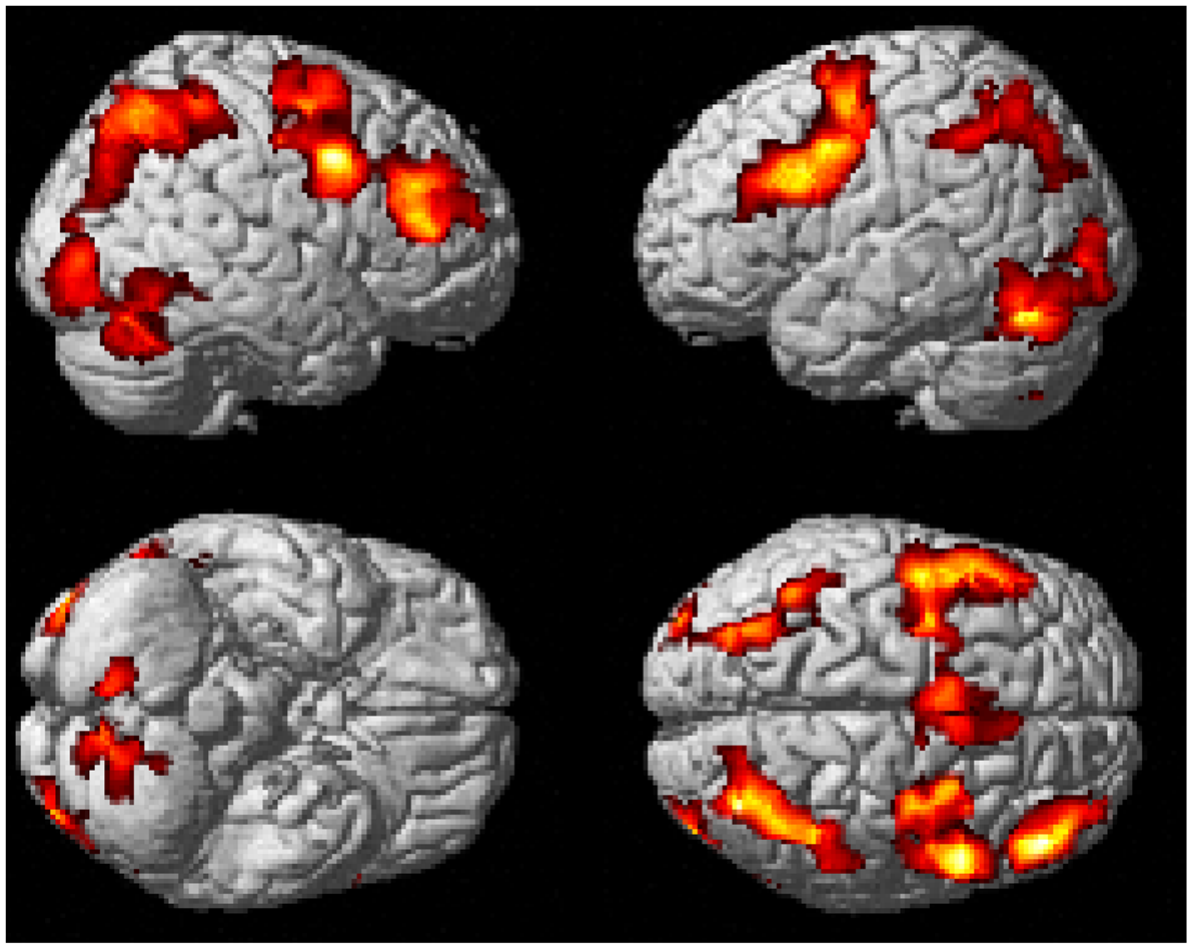
Brain regions active upon onset of ‘hit’ during the experimental task > control task contrast. Clusters were considered significant at a voxel height of p < .001 and cluster corrected at p < .05 (FWE corrected).

**Fig 8:**
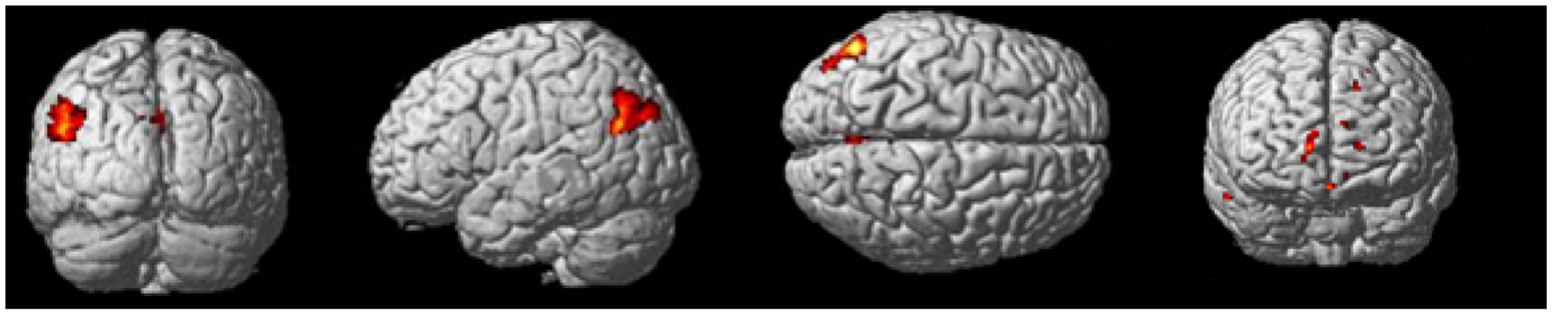
Brain regions active upon onset of ‘hit’ during the control task > experimental task contrast. Clusters were considered significant at a voxel height of p < .001 and cluster corrected at p < .05 (FWE corrected).

The hit (experimental) > hit (control) contrast revealed increased activation within cerebellum, occipital, inferior temporal areas, bilateral middle frontal gyrus and widespread parietal cortex. These regions can be seen in Table 7 and Fig 7. The reverse contrast however revealed differences in the right medial and left inferior region of the parietal lobes, in addition to bilateral activation in the orbital frontal lobe. These regions can be seen in Table 8 and Fig 8. There were no significant effects found between the experimental hits and the errors of commission.

**Table 7:** Clusters showing event-related differences between experimental task hits > control task hits (p = .001).

*Brain Region*	*Laterality*	*Cluster Size*	*Cluster Sig*.	*Peak t-score*	*MNI co-ords*
Middle Frontal Gyrus	R	3499	< .001	11.52	48 6 38
Middle Frontal Gyrus	L	3588	< .001	10.19	-44 2 38
Posterior Lobe	R	2057	< .001	9.02	24–66–30
Precuneus	R	2195	< .001	8.54	32–68 34
	L	2282	< .001	4.78	-14–70–46
Inferior Parietal Lobule	L	1247	< .001	4.12	-44–52 46

MNI = Montreal Neurological Institute,

B = Bilateral,

L = Left Hemisphere,

R = Right Hemisphere,

Cluster Sig. = Cluster significance.

**Table 8:** Clusters showing event-related differences between control task hits > experimental task hits (p = .001).

*Brain Region*	*Laterality*	*Cluster Size*	*Cluster Sig*.	*Peak t-score*	*MNI co-ords*
Medial Frontal Gyrus	R	382	.008	5.73	2 24–18
Precuneus	R	283	.027	5.18	4–60 30
Middle Temporal Gyrus	L	274	.030	5.12	-46–64 26

MNI = Montreal Neurological Institute,

B = Bilateral,

L = Left Hemisphere,

R = Right Hemisphere,

Cluster Sig. = Cluster significance.

A further analysis was performed on the event-related data using individual task performance as a covariate. Task performance was defined as number of hits on the experimental task and control task, entered separately for each participant, resulting in a 32x1 vector covariate. This analysis revealed no significant clusters between the hit (experimental) > hit (control) or the hit (control) > hit (experimental) contrasts when clusters were considered significant at a cluster forming threshold of t < .001 and cluster corrected at p < .05 (FWE corrected). Task performance was defined as number of hits on the experimental task and control task, entered separately for each participant, resulting in a 32x1 vector covariate.

#### Block and Event-Related Commonalities

**Fig 9:**
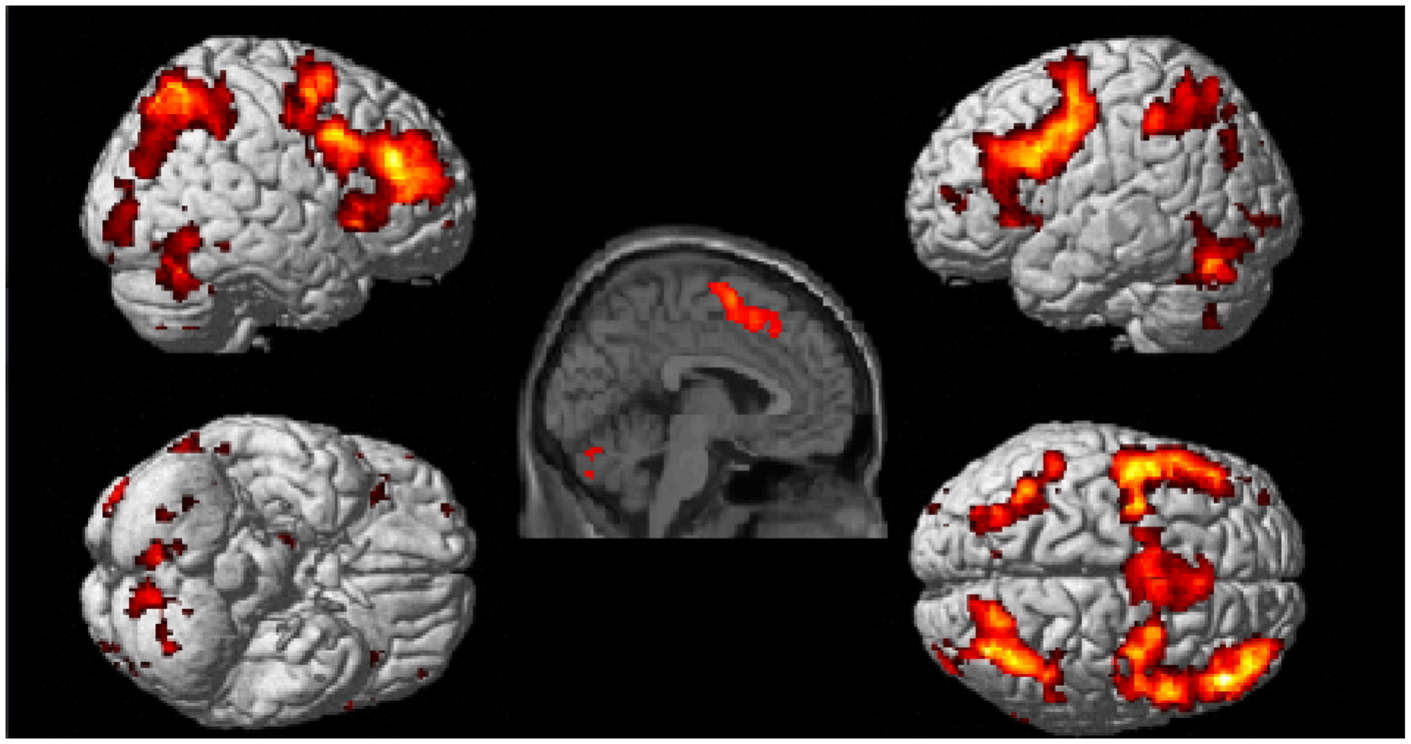
Common brain regions active between block analysis experimental > control task contrast and event-related experimental task hits > control task hits contrast. Cluster extent was considered significant at a voxel height of p < .001 and cluster extent corrected at p < .05 (FWE corrected).

**Fig 10:**
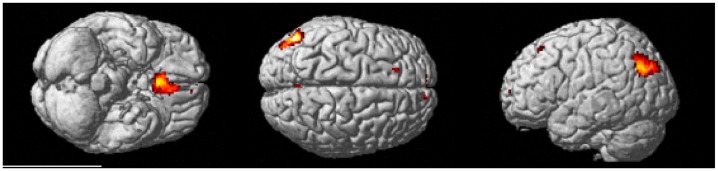
Common brain regions active between block analysis control task > experimental task contrast and event-related control task hits > experimental task hits contrast. Cluster extent was considered significant at a voxel height of p < .001 and cluster extent corrected at p < .05 (FWE corrected).

The contrast images produced from the block and event-related models were compared directly to assess commonalities between the two analyses. Whole brain analyses revealed seven clusters of brain activation that were significant between the block analysis experimental task > control contrast and event-related analysis experimental hits > control hits contrast (see Table 9 and Fig 9). A further three clusters were significant between the block analysis control task > experimental task contast and the event-related control hits > experimental task contrast (see Table 10 and Fig 10).

**Table 9:** Clusters showing similarities between block analysis experimental task > control task contrast and event-related analysis experimental task hits > control task hits (p = .001)

*Brain Region*	*Laterality*	*Cluster Size*	*Cluster Sig*.	*Peak t-score*	*MNI co-ords*
Superior Frontal Gyrus	R	6651	< .001	11.34	44 36 26
*Sub Region*: *Middle Frontal Gyrus*				8.93	52 6 36
Inferior Frontal Gyrus	L	3590	< .001	9.63	-44 8 30
*Sub Region*: *Precentral Gyrus*				9.14	-48 2 36
Precuneus	R	2417	< .001	8.84	32–66 34
Inferior Parietal Lobe	L	1365	< .001	6.66	-52–42 48
Cerebellum	R	768	< .001	5.53	28–62–30
Lentiform Nucleus	R	674	< .001	5.52	22–12 4
*Sub Region*: *Thalamus*				4.64	20–8 16
Cerebellum	L	1499	< .001	5.31	-30–66–22

MNI = Montreal Neurological Institute,

B = Bilateral,

L = Left Hemisphere,

R = Right Hemisphere,

Cluster Sig. = Cluster significance.

**Table 10:** Clusters showing similarities between block analysis control task > experimental task contrast and event-related analysis control task hits > experimental task hits (p = .001).

*Brain Region*	*Laterality*	*Cluster Size*	*Cluster Sig*.	*Peak t-score*	*MNI co-ords*
Precuneus	B	490	.003	7.55	-6–60 32
Superior Occipital Gyrus	L	421	.006	7.30	-42–84 36
*Sub Region*: *Middle Temporal Gyrus*				6.43	-50–78 32
Medial Frontal Gyrus	B	468	.004	4.77	2 24–18

MNI = Montreal Neurological Institute,

B = Bilateral,

L = Left Hemisphere,

R = Right Hemisphere,

Cluster Sig. = Cluster significance.

BOLD increases were observed bilaterally in frontal and parietal areas and cerebellar cortical areas. All cluster regions in the following tables refer to the area of peak activation.

## Discussion

The results show an expected difference in behavioural performance between the experimental and control tasks. Furthermore, there are clear distinctions with the brain activations that underpin each task type, shown both in the block and event-related analyses. An analysis for commonalities between analysis type show increases in the frontal lobe, precuenus and cerebellum associated with the experimental task.

The behavioural data presented here shows that participants responded at near ceiling level on the control task but at a significantly lower level on the experimental task. While this was expected, it appears that participants responded at a lower percentage (42.98%, 20.63 correct out of 48) correct level than in previous imaging research. This has shown levels of performance in normal subjects at 65% accuracy [14], 62% accuracy in a non-smoker control group [13], and 60% and 69% accuracy respectively in placebo groups [18, 19]. This could be accounted for by the fact that block lengths are shorter in this study than in the previous research, including neuroimaging studies, thus participants have less time to familiarise themselves with the task and there are fewer potential targets. On the other hand, participants were given adequate practice time prior to the start of the study and on each of the testing days prior to scanning. Alternatively, previous imaging research has typically used younger cohorts (aged between 18–35 years) which may suggest that the older cohort used in this study were less able to cope with the cognitive demands of the experimental task. An interesting future direction would be to directly compare a variety of age groups across the same experimental design to ascertain an index of cognitive ability throughout the age spectrum from children through to old age.

The low performance rate does make interpretation of the results problematic as there are less hits than expected thus increasing noise in the fMRI data. Future research should be directed towards direct comparison of different age groups, such as current studies into the effects of nutraceuticals on RVIP performance [26].

Reaction times were significantly increased in the experimental task compared with the control task. Again, this was expected given the relative ease of the control task meaning that participants can respond freely without the cognitive load arising from directing attentional resources to the experimental task. This is supported by the fact there were no false positives recorded in the control condition. This also suggests that the participants were well trained on the task given that no participant pressed the response button in error during any of the control task number streams in the six blocks across the two individual scanning runs per scanning session (six minutes total). The errors of commission in the experimental task are unsurprising given the relative difficulty of the task, supported by the mean results that show participants’ performance did not approach ceiling level.

When comparing both the experimental and control tasks against the baseline, the results suggest that participants are not challenged by the control task. There are only two clusters of significant activation which are limited to the occipital lobe and the inferior temporal gyrus. The experimental task however suggests that participants require an extensive network of activation to successfully complete the task. These regions are similar to those shown in previous studies when comparing experimental and control task activation, such as the prefrontal cortex and parietal activations. The contrast estimates show that there appears to be increased activation in regions associated with successful task completion in the experimental task > baseline condition. However, there is shown to be decreases in activation within the same regions in the control task > baseline condition. This suggests deactivation of task-relevant regions during the control task, presumably reflecting the need for these areas for successful completion of the experimental task.

When compared with the control task in the block analysis, the experimental task was associated with activation of a network of frontal, parietal, cerebellar and occipital regions similar to those shown in previous studies [14, 18]. The largest volume of clusters was shown in the cerebellum and middle frontal gyrus (but all clusters were highly significant).

The activation of the precuneus during the experimental task is in keeping with Lawrence et al (21), though this is not discussed other than in the context of the precuneus’ location as part of the parietal lobule. A definitive review of the precuneus by Cavanna and Trimble [27] presented the seemingly important role it plays in visuo-spatial imagery, episodic memory and self-processing as well as highlighting the cortical and subcortical connections. The precuneus is also said to be a part of the default mode network (DMN). The theory, history and current research into the DMN is beyond the scope of this paper, but briefly, the DMN refers to a network of brain regions that become active during ‘rest’ or at least when participants are not engaging in a particular task per se, as they would tend to do in a neuroimaging environment [28–31]. Margulies et al [32] showed that the precuneus may be divided into specific regions with connections relating to the visual system, sensorimotor activity and, importantly for the current study, a region associated with cognitive processing. Research has shown that atrophy in the precuneus correlates with early onset Alzheimer’s Disease [33] and is supported by Buckner et al [34] who showed that when cortical networks involving the precuneus are damaged, memory and executive function is compromised. There is also the suggestion that long-range cortical connections between frontal regions and the precuneus play a role in executive function, supported by research showing that ADHD patients show less functional connectivity between these areas when compared with healthy controls [35]. Given that the RVIP task requires some level of executive control to both identify a target and reject non-targets, perhaps the precuneus plays an important role in this element of successful task completion, perhaps due to connectivity to frontal regions shown to be active during the experimental task as well as during the control task where the precuneus was the peak of the largest cluster in the commonality analysis between the control hits and control task contrast vs. the experimental task.

The precuneus activation appears to be the peak activation in a cluster that extends across the intraparietal sulcus (IPS). The IPS has been traditionally viewed as an interface between the visual world and appropriate planning and execution of movement responses [36]. There is however research suggesting the IPS is involved in attention and working memory tasks [37–39] which may suggest this is why the IPS is active in the present study. Indeed, the IPS is included in the postulated task-positive network [40] which is a network of regions that may become active during attentionally demanding cognitive tasks. The activation of the IPS in this context correlates with the increase in cognitive demand between the experimental task compared with the control task.

The prefrontal activation comprising of the middle, superior and inferior frontal gyri in the experimental task when compared with the control task was expected based on previous RVIP imaging research (Lawrence et al 2002) (20). The prefrontal cortex is implicated in a range of cognitive processing. The middle frontal gyrus has been implicated in monitoring performance-based information during cognitive processing, such as response errors and response uncertainty [41]. Recruitment of such a system may be necessary given the higher cognitive load in the experimental task compared with the control task. The left middle frontal gyrus also showed the second largest cluster size after the cerebellar activation. The activation of the dorsolateral prefrontal cortex (DLPFC) is not unexpected in this study given the important role it plays in working memory as shown by fMRI [42, 43], repetitive transcranial magnetic stimulation (rTMS) [44] and PET [45, 46]. Interestingly, there is research which suggests that dorsal pathways feeding forward from the parietal cortex terminate at the DLPFC, determining the correct response to a particular cognitive challenge [47].

The right lentiform nucleus is part of the basal ganglia, comprising of the putamen and globus pallidus located in the dorsal striatum. Both structures are implicated in the control and preparation of voluntary movement. Interestingly, recent research has suggested that this activation maybe driven by the frontal lobes [48]. Participants underwent an fMRI scan, including a switching task known to activate the putamen, before and after offline rTMS over the frontal cortex. Post TMS scans resulted in disrupted expected task related activation from the putamen while also decreasing connectivity between the frontal and striatal regions. This suggests a functional connection between the frontal cortex and striatum worthy of further research. Recent research in animal models has shown increased theta coherence during attentive wakefulness, suggesting that the striatal regions play an important role in integrating multiple systems involved in cognition and motor systems [49].

As discussed, previous neuroimaging studies comparing block activation of the experimental and control tasks show increases in frontal and cerebellar activation in young cohorts. The middle-age cohort used here appears to show similar changes in activation between the experimental tasks, as well as increases in activation in the lentiform nucleus, for example, that haven’t been shown in previous studies. Perhaps then an interesting future direction would be to develop a direct comparison study between young, middle aged and even old age cohorts to potentially map the changes in activation in time associated with this particular attention task.

The block-by-block comparison of activation within the individual experimental and control blocks showed no significant differences. This suggests that the regions recruited for successful processing of the task did not change over time, nor was the recruitment of other regions necessary. As mentioned previously with the behavioural data, the length of the task block and the rest periods between task blocks may have been sufficient enough to allow recovery and thus prolonging performance by preventing fatigue effects sometimes manifested by decreased performance over time in the classic version of the task which can last between five to ten minutes.

The event-related analysis reveals a similar network of brain regions involved in correct responses to stimuli in the experimental task when compared against correct responses during the control task. Indeed, commonalities between the block and event related analyses were investigated and showed similar activation, especially in medial and inferior frontal activation as well as precuneus and cerebellum activation. These commonalities are, again, similar to those shown in previous RVIP neuroimaging research (20).

This study is the first to date to employ an event-related analysis on the RVIP task showing a fronto-parietal network of activation. That there were no differences between the target hits and errors of commission is perhaps not surprising given the small number of events by the errors of commission compared with target hits. *Furthermore*, *there were no further differences in activation when using performance as a covariate in the hit (experimental) > hit (control) nor the hit (control) > hit (experimental) conditions*. *This suggests that the brain activation differences shown correlate with task difficulty*. *That the performance covariate doesn’t expand on this may suggest that task differences are already shown irrespective of the performance covariate*. An interesting finding was that both the right inferior frontal gyrus (rIFG) and the lentiform nucleus showed activation in the experimental task during the block analysis which was not evident in the event-related analysis. This could be explained by these regions’ role in response inhibition [50–52]. During the experimental block, it is possible that the increased rIFG and lentiform nucleus BOLD response is due to monitoring the digit stream and inhibiting incorrect responses. That this activation is not shown in the event-related analysis is unsurprising given that the events in question are from a correct response, therefore there is no need for inappropriate response inhibition.

In both the block and event related analyses, the control > experimental contrast showed increased activation in the inferior parietal lobe with the peak activation in the middle temporal gyrus as well as cuneus in the block analysis only. The inferior parietal lobe has been implicated as part of the DMN previously (36–39) while the cuneus may play a role as a hub between DMN and frontal regions [53]. That these regions are becoming active during the control task suggests that the task does not have a high level of cognitive demand and participants’ engagement with the task is smaller. There is increased activation in a small cluster of the medial frontal gyrus shown in the event-related analysis in the control task which is located in a different spatial location from the middle frontal gyri activation shown in the experimental task. It is likely that this region is playing a role in the monitoring of events and responses, as described above, but the level of cognitive effort required is much lower than during the experimental task, hence the relatively small cluster size compared with the activation seen during the experimental task.

Traditionally, the RVIP task utilises sustained attention over a long duration of time—typically in the region of five to ten minutes. A limitation of this study, which can also be levelled at previous RVIP functional imaging research, is the shorter duration of blocks used due to minimising fatigue in the scanner environment. However, whilst using sixty second blocks may not reflect the levels of demand of the original task, short blocks are necessary for obtaining meaningful data from the hemodynamic response function (HRF) that is used in BOLD research. Perhaps it is more appropriate to cite the imaging paradigms of the RVIP task as tasks of pure attention rather than tasks of sustained attention. Further to this, a meta-analysis of correlates of working memory [54] complied results of 189 fMRI studies investigating working memory and showed fronto-parietal activation similar to that shown in the block experimental task > control task shown above. Given these similarities, there appears to be a working memory component which is required for successful task completion in this particular version of the RVIP task. This is a particularly useful that there are similar findings in this meta-analysis given the small sample size of the cohort in this research.

It would be interesting for future research to use a cross-over design and undertake the RVIP task under varying block durations and assess any differences in the regions that are active or behavioural performance. Of course, lengthening block durations may also impact on the resources given the subsequent increases in imaging time.

Using shorter block durations reduced the numbers of targets in both the experimental and control tasks. As a result, this leads to a reduced total number of hits when compared with previous imaging, and indeed behavioural, research. Using two functional runs during each scan session went some way to addressing this issue. The hit-rate in this study was lower than previous imaging studies (in terms of percentage accuracy). As previously suggested, this could be due to the age of the cohort used in the present study. Perhaps then the reduced hit-rate shown here is indicative of normal cognitive aging. Furthermore, adding more task blocks or lengthening task blocks to achieve a greater number of hits and errors of commission may be beneficial to increase the statistical power of the event-related models presented here.

It appears that the RVIP task, with the paradigm described in this paper, is sensitive to cognitive demand. The next stage of the research should be concerned with replicating these findings, perhaps with a larger cohort. Recent published work showed this version of the RVIP task to be sensitive to change after acute administration of nutraceutical preparations in healthy adult participants (49). This paper also begins to establishing an event related approach to analysing RVIP research in future studies. Future studies may wish to consider an analysis of the data that includes modelling the events of ‘no interest’ such as two consecutive odd numbers followed by an even or vice versa. This may be interesting as researchers could potentially model the stages of attention required for successful task completion and the activation associated with initial alertness to a stimuli (for example an odd number), followed by activation associated with preparation for a potential response (two odd numbers in succession) and then the resetting of this system when viewing an even number. Unfortunately, this analysis was beyond the scope of the present study.

To conclude, the version of the RVIP task presented here is sensitive to cognitive demand as shown by the statistically significant differences in performance between the cognitively demanding experimental task and the less demanding control task. The data presented also shows an event-related analysis of functional imaging in the RVIP task for the first time, as far as the authors are aware. The results show regions involved specifically in the overall processing of the task as shown by the block analysis as well as those regions involved in the phasic aspects of the task, such as hits or errors of commission. Frontal gyri, parietal regions and the cerebellum are shown to be active in both the block and event-related analyses, implying their importance in sustained attention and successful completion of this particular attention task. The combined analyses utilised here show that there are benefits to using a combined analysis protocol in future RVIP research, which in turn may lead to a new applications of this particular attention task.
